# Characteristics of COVID-19 infection and antibody formation in patients known at a tertiary immunology department

**DOI:** 10.1016/j.jtauto.2021.100084

**Published:** 2021-01-29

**Authors:** Niels A.D. Guchelaar, Jan A.M. van Laar, Maud A.W. Hermans, Tim B. van der Houwen, Sibel Atmaca, Maurits S. van Maaren, Zana Brkic, Paul L.A. van Daele, Virgil A.S.H. Dalm, P. Martin van Hagen, Saskia M. Rombach

**Affiliations:** Department of Internal Medicine, Division of Allergy and Clinical Immunology, Academic Center for Rare Systemic Immune Diseases, Erasmus MC University Medical Center, Rotterdam, the Netherlands

**Keywords:** COVID-19, Immunologic deficiency syndromes, Immunocompromised, Immunosuppressive agents, Antibodies

## Abstract

**Background:**

Knowledge about COVID-19 infections is expanding, although knowledge about the disease course and antibody formation in patients with an auto-immune disease or immunodeficiency is not fully unraveled yet. It could be hypothesized that immunodeficient patients, due to immunosuppressive drugs or their disease, have a more severe disease course due to their immunocompromised state. However, it could also be hypothesized that some of the immunosuppressive drugs protect against a hyperinflammatory state.

**Methods:**

We collected data on the incidence of COVID-19, disease course and SARS-CoV-2 antibody formation in COVID-19 positive patients in a cohort of patients (n ​= ​4497) known at the Clinical Immunology outpatient clinic in a tertiary care hospital in the Netherlands.

**Results:**

In the first six months of the pandemic, 16 patients were identified with COVID-19, 14 by nasal swab PCR, and 2 patients by SARS-CoV-2 antibodies. Eight patients were admitted to the hospital. SARS-CoV-2 antibodies were measured in 8 patients and were detectable in all, including one patient on B-cell ablative therapy and one patient with Common Variable Immunodeficiency Disorder.

**Conclusion:**

This study indicates that the disease course differs among immunocompromised patients, independently of (dis)continuation of immunosuppressive drugs. Antibody production for SARS-CoV-2 in immunocompromised patients was shown. More research needs to be conducted to confirm these observations and guidelines regarding (dis)continuation of immunosuppressive drugs in COVID-19 positive immunocompromised patients should be developed.

## Highlights

• The disease course of COVID-19 largely differs among immunocompromised individuals.
• Antibody production against SARS-CoV-2 is noticed in immunocompromised patients.
• Further recommendations on (dis)continuation on immunosuppressive drugs during a COVID-19 infection are needed.

## 1 Introduction

An infection with SARS-CoV-2 causes symptoms of the respiratory tract, but increasing evidence shows that almost every organ system can be involved [[1], [2]]. In some patients, the disease course can be complicated by a potentially fatal cytokine-driven hyperinflammatory response [[3], [4], [5]]. It may be suggested that immunocompromised patients, either due to a primary immunodeficiency or a secondary immunodeficiency caused by the usage of immunosuppressive drugs, are at increased risk for infection and a more severe disease course with SARS-CoV-2 [6]. Conclusive data on this subject are missing, however. On the other hand, specific immunosuppressive drugs are used in the treatment of the hyperinflammatory state [[7], [8], [9]]. It could therefore be hypothesized that anti-cytokine therapy could mask the symptoms of an infection with COVID-19 or alter the disease course. Furthermore, there are not much data on the antibody production of SARS-CoV-2 in immunocompromised patients. To delineate the effect of an underlying immunological condition and/or immunosuppression on the course of COVID-19, we performed a descriptive study to investigate the incidence, disease course and SARS-CoV-2 antibody production in a cohort of patients with a primary or secondary immunodeficiency. For this study, approval from the medical ethical committee was requested and obtained.

## 2 Results and discussion

Our cohort consists of 4497 patients that are attending the outpatient clinic of the department of Clinical Immunology at the Erasmus University Medical Center (Rotterdam, the Netherlands). From the start of the pandemic in the Netherlands, at the end of February 2020, data from the patients known at the Clinical Immunology clinic referred to the emergency department and/or being admitted at the ward or ICU because of (a suspicion of) COVID-19 were collected prospectively. In addition, all patients at the immunology outpatient clinic, with auto-immune, auto-inflammatory and primary immunodeficiency diseases, are instructed to contact the Clinical Immunology department when they have symptoms of an infection. From the start of the COVID-19 epidemic in the Netherlands patients were questioned about potential COVID-19 symptoms in the outpatient clinic, and when admitted elsewhere correspondences from other hospitals were collected. Data on clinical features and use of immunosuppressive drugs in patients with COVID-19 known at the Clinical Immunology department were analyzed in the first six months of the epidemic until August 2020. Furthermore, the incidence of COVID-19 in our cohort was investigated. A total of 67 patients in our cohort were tested for SARS-CoV-2 by nasopharyngeal swab, of whom 14 tested positive for COVID-19 infection (21%) (Table 1). Two patients (patient 10 and 13) had typical COVID-19 symptoms, but did not have a PCR-test at the time of symptoms. Afterwards these patients demonstrated serum SARS-CoV-2 antibodies.

**Table 1:** Clinical features of the COVID-19 positive patients.

Patient, age/gender	Admission	Diagnosis	IgM	Ig	Duration (D)	BMI (kg/m^2^)	Symptoms	ISD
1, 40/F	Home	CVID	1	1	41 days	24.2	Cough, ST, fever, dyspnea, chest pain, sinusitis	–
2, 21/F	GW, 9 days	BD	1	1	18 days	27	CC, cough, fever, dyspnea, diarrhea	Colchicine, prednisone, IFX^a^, dapsone
3, 46/F	GW, 19 days ICU, 29 days MV	NMO	1	1	56 days	34	CC, fever, dyspnea	Mycophenolic acid^a^ Rituximab (10 months earlier)
4, 63/F	GW, 17 days	Sarcoidosis	1	1	60 days	34	Emesis, ST, chest pain, fever	HCQ, MTX^a^
5, 64/F	NH	SADNI	x	x	16 days	25.1	Fever	HyQvia
6, 50/F	GW, 7 days	ASS	1	x	74 days	35	Fever, cough, shoulder blade pain	–
7, 91/M	NH, dec.	Chronic urticaria	x	x	Unknown	28.2	Unknown	Dexamethasone
8, 85/M	GW, 7 days, dec.	Secondary IMD	x	x	10 days	22.4	Cough, fever, dyspnea	IgG suppletion
9, 33/F	Home	Polymyositis	1	1	50 days	20.4	CC, chest pain	Rituximab (3 months earlier)
10, 51/F	Home	SS with PH	1	1	31 days	30.2	Emesis, diarrhea, headache, chest pain, LOTS	–
11, 54/M	ICU, 21, days, no MV	BD, NPC	x	x	21 days	29.1	Pneumonia	Colchicine, Azathioprine, chemotherapy
12, 49/F	ICU, 6 days, MV	BD	x	x	25 days	31.9	Dyspnea, headache, cough	Colchicine^a^
13, 54/F	Home	Sarcoidosis	1	1	Unknown	39.2	Fever, chills	MTX^a^
14, 34/F	Home	BD	x	x	14 days	19.2	Headache, emesis, dyspnea	Azathioprine^a^
15, 57/M	GW, 8 days	RP	x	x	8 days	26.8	Fever	MTX^a^
16, 28/M	Home	BD	x	x	19 days	21	CC, ST, headache	Apremilast

ASS ​= ​Anti-synthetase syndrome, BD = Behçet’s disease, BMI = Body Mass Index (in kg/m^2^), CC ​= ​common cold, CVID = Common Variable Immunodeficiency Disorder, D ​= ​the duration of illness defined as the amount of days between the first day of reported symptoms and the last day of reported symptoms, dec. ​= ​deceased, F ​= ​female, GW ​= ​general ward, HCQ ​= ​hydroxychloroquine, IC = Intensive Care, IFX ​= ​infliximab, Ig ​= ​total Ig-SARS-CoV-2, IgM ​= ​IgM-SARS-CoV-2, IMD ​= ​immunodeficiency, ISD ​= ​immunosuppressive drugs, LOTS ​= ​loss of sense of taste and smell, M ​= ​male, MTX ​= ​methotrexate, NH = Nursing Home, NMO ​= ​aguaporine-4 associated neuromyelitis optica, NPC = Nasopharyngeal carcinoma, MV ​= ​mechanical ventilation, PH ​= ​pulmonary hypertension, RP ​= ​Relapsing polychondritis, SADNI = Selective Antibody Deficiency with Normal Immunoglobulins, SS ​= ​systemic sclerosis, ST ​= ​sore throat, x ​= ​not tested, 1=positive.

a = ​discontinued upon positive COVID-19 test.

The clinical symptoms differed significantly among patients. Some patients only presented with fever, or minor symptoms, whereas other patients presented with severe respiratory symptoms. Half of the patients (8/16) were admitted to the hospital and 3 of them were admitted to the Intensive Care Unit (ICU). A mortality rate of 2/16 was reported. Both deaths concerned patients of 85 years and above. One patient used dexamethasone for chronic spontaneous urticaria and the other immunoglobulin substitution therapy for a secondary immunodeficiency due to COPD. The incidence of SARS-CoV-2 by nasal swab PCR in this cohort is 14/4497 ​= ​0.31% in the first six months, compared to the incidence of 67543/17 ​445 ​447 ​= ​0.39% in the first six months in the general population of the Netherlands in that period [10,11].

Antibodies were measured in 8 patients in total, in 6 patients who also had a positive PCR-SARS-CoV-2 test. In a subgroup of patients antibodies were not measured because patients had not attended the out-patient clinic yet. In 5/6 PCR-positive patients, both the isotypes IgM and total Ig were detectable. One patient, with a very recent infection, only showed positive IgM antibodies. Of those 6 SARS-CoV-2 positive patients, 5 patients used immunosuppressive drugs at the time of infection with COVID-19. Immunosuppressive drugs were discontinued in 4 of them during the disease course (Table 1). Patient 9 was treated with rituximab (B-cell ablative therapy) three months before infection because of polymyositis. Rituximab targets the CD20 antigen and the effect lasts for approximately 3–8 months [12]. Remarkably, both IgM and total Ig antibodies, measured four weeks after the first signs of COVID-19, were positive. By using FACS analysis no B-cells could be detected in peripheral blood of this patient, although plasmablasts were still detectable in peripheral blood. No admission to the hospital was needed and she recovered well at home. This case unexpectedly illustrates that patients using immunosuppressive drugs, even immunosuppressive drugs targeting the B-cells, still can produce antibodies against SARS-CoV-2. COVID-19 antibodies were also seen in our cohort in patients with non-B cell ablative immunosuppressive drugs.

A total of 2 patients were diagnosed with a primary immunodeficiency. Patient 1 was known with Common Variable Immunodeficiency Disorder (CVID), an primary immunodeficiency characterized by an increased risk of infections and deficit of antibody production due to an impaired B-cell differentiation [13]. She was diagnosed with CVID 1 year ago and had not yet started with immunoglobulin substitution. She presented with mild respiratory symptoms and fever. The PCR-test for SARS-CoV-2 was positive and she fully recovered at home. She was described in an international study [14], where she had a less severe disease course than other patients, but antibodies were not measured then. Both IgM-SARS-CoV-2 and total Ig-SARS-CoV-2, measured seven weeks after the first symptoms, were positive despite an impaired B-cell function. Patient 5 was known with Selective Antibody Deficiency with Normal Immunoglobulins (SADNI). She was already being treated with immunoglobulins and presented with fever as the only symptom and recovered well without other treatments.

In this observational cohort we studied the characteristics and antibody formation in COVID-19 positive patients and the incidence of COVID-19 in patients known at the immunology department in the first six months of the pandemic. Out of the total cohort of 4479 outpatients, known at the outpatient clinic, sixty-seven patients were tested for COVID-19 by nasal swab PCR, of which fourteen (21%) had COVID-19. Initially, test capacity in the Netherlands was limited and was only restricted to patients with more severe symptoms who were attending the hospital. At a later stage, only patients that showed clinical symptoms of an infection were allowed to be tested. As a consequence, patients who were asymptomatic or who did not report symptoms might have been missed. The reported incidence could therefore be an underestimation. Moreover, the cohort was not screened systemically, which could also cause underestimation of the incidence and a overestimation of the percentage of admission. However, this study suggests that the incidence of a COVID-19 infection in immunocompromised patients does not seem to be increased compared to the general population. This was also reported by others at the beginning of the pandemic [15]. Whether this is a result of strict adherence to quarantine measures, or due to an underestimation because not all patients might have reported their COVID-19 symptoms, cannot be answered by this study. A larger systematical study should be conducted to determine the incidence more accurately in this patient population. A relatively large proportion of the COVID-19 positive patients had to be admitted to the hospital (50%), both patients who continued the immunosuppressive drugs as well as patients who discontinued these drugs. The proportion of admissions in the general population was 18% [11]. Furthermore, 3/16 (19%) patients were admitted to the ICU. This would suggest that the disease course is more protracted in immunocompromised patients, however the number of COVID-19 infections is too small to draw any definitive conclusions. In addition, if the incidence of COVID-19 is actually higher because of underestimation of the number of mild cases staying at home, the proportion of patients being admitted would be lower. Previously, it has been reported that the admission rate in patients with rheumatic diseases was 44% and was similar to those without rheumatic diseases, but patients with a rheumatic disease did require ICU and mechanical ventilation more often [16]. In the present study the number of ICU admissions was not as high as reported previously, but seems higher than in the general population [16]. In a few patients the duration of disease was quite long. This is however fully based on whether patients still reported symptoms of COVID-19, even minor. This is also reflected by the duration patients were admitted, which is much shorter than the duration of reported symptoms in most of them. Whether the disease duration is prolonged in immunocompromised patients deserves further study.

At the moment, there is no universal guideline about the continuation of immunosuppressive drugs during a COVID-19 infection [17]. This decision should be made for each patient individually. The number of patients in this study was too small to determine the exact effect of immunosuppressive drugs on the disease course. A meta-analysis showed a more protracted course of a COVID-19 infection in patients using disease modifying antirheumatic drugs (DMARDs) or DMARD combination therapy with a biological or targeted DMARD therapy, while biological monotherapy, including anti-TNF monotherapy reduced the risk of a severe COVID-19 infection [18]. Further studies are needed to confirm this effect of immunosuppressive drugs in a COVID-19 infection.

An unexpected finding is the production of SARS-CoV-2 antibodies in patients with either primary B-cell dysfunction or due to B-cell ablative therapy. The protection against a new infection and the half-time of these antibodies is not yet investigated, and repeated antibody measurement in time can provide more insight in to this.

## Credit author statement

Each named author has substantially contributed to conducting the underlying research and drafting this manuscript. Additionally, authors have no conflict of interest, financial or otherwise.

## Declaration of competing interest

The authors declare that they have no known competing financial interests or personal relationships that could have appeared to influence the work reported in this paper.
